# Consistently Sampled Correlation Filters with Space Anisotropic Regularization for Visual Tracking

**DOI:** 10.3390/s17122889

**Published:** 2017-12-12

**Authors:** Guokai Shi, Tingfa Xu, Jie Guo, Jiqiang Luo, Yuankun Li

**Affiliations:** 1 School of Optoelectronics, Image Engineering & Video Technology Lab, Beijing Institute of Technology, Beijing 100081, China; shi_guokai_123@126.com (G.S.); jieguo_2013@163.com (J.G.); luojiqiang@yeah.net (J.L.); liyuankunbixian@gmail.com (Y.L.); 2 Key Laboratory of Photoelectronic Imaging Technology and System, Ministry of Education of China, Beijing 100081, China

**Keywords:** correlation filter, online learning, sample consistency, visual tracking

## Abstract

Most existing correlation filter-based tracking algorithms, which use fixed patches and cyclic shifts as training and detection measures, assume that the training samples are reliable and ignore the inconsistencies between training samples and detection samples. We propose to construct and study a consistently sampled correlation filter with space anisotropic regularization (CSSAR) to solve these two problems simultaneously. Our approach constructs a spatiotemporally consistent sample strategy to alleviate the redundancies in training samples caused by the cyclical shifts, eliminate the inconsistencies between training samples and detection samples, and introduce space anisotropic regularization to constrain the correlation filter for alleviating drift caused by occlusion. Moreover, an optimization strategy based on the Gauss-Seidel method was developed for obtaining robust and efficient online learning. Both qualitative and quantitative evaluations demonstrate that our tracker outperforms state-of-the-art trackers in object tracking benchmarks (OTBs).

## 1. Introduction

Visual tracking plays an important role in computer vision and has been widely used in many smart and automatic systems in recent years. During tracking, the appearance of the target has random variation and is unpredictable. This is a very challenging issue because prior information on target appearance is inadequate. Therefore, a robust tracker must have a good generalization ability as to adapt to the significant variability in the appearance of targets. It is difficult to construct a robust tracking model that can adapt to changes in the appearance of targets.

Recent research into visual tracking has focused mainly on the appearance model, which can be broadly divided into two categories: generative models and discriminative models. Generative models search for the closest description in the model space to the observed target in order to estimate target state. Because these models adopt an appearance model to describe the target appearance state without considering background information on the target, they have low discriminative ability if the scene is complex. Compared with generative models, discriminative models, which discriminate the target from the background by learning through an online appearance classifier, have better discrimination and generalization ability to respond to changes in the appearance of targets.

Recently, discriminative correlation filter (DCF) tracking models have shown significant advantages both in accuracy and speed compared with traditional discriminative trackers. DCF-based trackers are different from traditional discriminative trackers in both learning and detection. During the process of learning, training samples use continuous labels instead of positive and negative labels. These continuous labels include positional information on the target, and it is assumed that the labels obey a two-dimensional single-centered Gaussian distribution. Therefore, DCF filters have a higher positioning capability. Similarly, for detection, candidate target regions are cropped from a loop which shifts from the last estimated target position. It is different from traditional discriminative tracking models where candidate target regions are selected randomly. The positioning ability of traditional discriminative trackers is limited by the randomness of candidate target regions, while DCF-based trackers avoid this limitation and so have a more accurate positioning ability. In addition, DCF-based trackers use dense sampling while traditional discriminative trackers use random sampling. In dense sampling, all samples can be aligned into a circulant matrix. The circulant matrix can then be transformed into a diagonal matrix in the Discrete Fourier Transform (DFT), which can significantly improve the computation speed of the tracking model.

The performance of DCF-based trackers benefits from dense sampling and continuous sample labels. However, the establishment of these two strategies (the dense sampling and the continuous sample labels) is based on some assumptions. Firstly, the samples obtained by dense sampling are only approximations of reality. Approximations are valid only when the background is relatively smooth, which means that the sampling method is unreliable if there are cluttered features or similar targets in the background. Secondly, sample labels obey single-centered Gaussian distribution, which means that the center of the distribution corresponds to the real target. However, in actual tracking, this assumption is untenable because of the existence of motion-blurred targets or occluded targets.

In order to overcome the above problems and make a more generalizable tracker, we propose a new DCF-based tracker. Our main contributions are as follows:

(1) We propose a new DCF-based tracking model which integrates two strategies (anisotropic spatially-regularized constraints and consistent sampling) into a unified DCF-based tracking model.
(2) We propose to study an anisotropic spatially-regularized filter, which is used to penalize the response of occluded areas of the target.
(3) We propose to use a spatially-weighted function for every training and detection sample. This strategy can alleviate redundancies in training samples caused by cyclical shifts and eliminate inconsistencies between training samples and detection samples.
(4) We propose to further develop an optimization strategy including a closed-form solution and an iterative method. The iterative method is based on the Gauss-Seidel method which can make online learning robust and efficient.

The rest of the paper is organized as follows: In Section 2, we introduce related works on visual tracking. The optimization of the unified objective function and the tracking algorithm are presented in Section 3. Quantitative and qualitative experiments comparing our method with other state-of-the-art methods are discussed in Section 4. In Section 5, we report the conclusions of our paper.

## 2. Related Works

Various trackers, which include appearance models, motion models, and search strategies, have been proposed in visual field tracking. Our contributions are focused on the appearance model. The appearance model can be divided into three categories: generative models, discriminative models, and DCF models. In this section, we will briefly list the most representative trackers in the three categories and discuss the works that are most relevant to the proposed tracker.

### 2.1. Generative Trackers

A generative tracker learns target appearance model to dynamically represent a set of target observations. Therefore, the key to generative tracking models is to find the most similar candidate when target appearance features have undergone some changes in an image sequence. Up to now, there have been many representative generative models, e.g., mean shift based tracking [1], incremental visual tracking (IVT) [2], fragment-based tracking (Frag) [3], multi-task based tracking (MTT) [4,5], low-rank sparse based tracking [6], tracking based on exclusive context modelling [7], and sparse structural learning-based tracking [8,9]. The main drawbacks of these methods are reflected in two aspects. First, the performance of a model is limited by the dimensions of the model representation space. Second, these models don’t utilize the background information of the target effectively and so show less discrimination when the scene is more complex.

### 2.2. Discriminative Trackers

Compared with generative models, discriminative models, which take full advantage of the differences between the target and the background, have better discrimination and generalization ability as to cover a wide range of appearance changes. Traditional discriminative trackers formulate visual object tracking as a binary classification problem that searches for the target location most distinctive from the background. Common discriminative trackers, which have a large influence on visual tracking, include the multiple instance learning tracking model (MIL) [10], the online adaptive boosting tracking model (OAB) [11], the support vector tracking model (SVM) [12], the tracking-learning-detection model (TLD) [13], online random forests tracking (ORF) [14], a tracker based on transfer learning with Gaussian processes regression (TGPR) [15], and multi-expert entropy minimization (MEEM) [16]. The key challenge for discriminative trackers is the sampling of training samples online and adapting to updates in the target appearance model. Most discriminative models, which are binary classifiers and do not include positional information on the target in training samples and candidate tests, can’t directly estimate the specific location of the target. Additionally, the accuracy of target localization is limited by the number of candidate tests and sampling randomness.

### 2.3. DCF-Based Trackers

DCF-based trackers overcome the shortcomings of generative and discriminative models and they exhibit promising results for visual tracking. Initially, the minimum output sum of squared error (MOSSE) [17] successfully used correlation filters in visual tracking and showed promising speed, however, the feature used in the model is restricted to a single channel, so its performance is not as promising as its speed. Subsequently, the DCF-based tracking framework is developed to incorporate multi-channel feature maps [18,19], which allows high-dimensional features to be used for improved tracking. Since then, the importance of multi-channel feature maps has attracted much attention to DCF-based tracking. For example, Liu et al. [20] used multiple kernelized correlation filter trackers to represent different parts of the object and track them jointly, alleviating serious occlusion. Discriminative scale space tracking (DSST) [21], which utilizes one-dimensional correlation filters for online estimation of target scale, is proposed to overcome a wide range of changes to target scale. Long-term correlation tracking (LCT) [22] is proposed to add random fern detection into the DCF-based tracking framework for alleviating serious occlusion. In [23], complementary learners tracking, which integrates the advantages of DSST [21] and DAT [24] effectively, is proposed by Bertinetto et al. to alleviate large deformations in target appearance. In [25], Gundogdu et al. proposed to develop a Convolutional Neural Network (CNN) model for circularly correcting the correlation filter and the observation, and the improve the resulting correlation output. In [26], Danelljan et al. proposed to study continuous convolution operators for visual tracking (C-COT), which enables for efficient integration of multi-resolution deep features. In [27], Danelljan et al. proposed efficient convolution operators for visual tracking (ECO), which is an improvement on C-COT, and successfully tackles the causes of over-fitting and gets better generalization of the target appearance.

Recently, some tracking algorithms have focused on addressing the boundary effects that are most relevant to our work. Galoogahi et al. [28] proposed a Background-Aware CF (BACF) that can efficiently model how both the foreground and background of the object varies over time. BACF exploits all background patches as negative examples for learning a filter and so is more discriminative against background clutter. However, BACF does not take into account the reliability of positive samples: if the target is occluded, positive samples contain some background information and so may lead to drifts in subsequent tracking. In [29], Lukežič et al. introduced the channel and spatial reliability concepts to DCF tracking (CSR-DCF). An automatically estimated spatial reliability map restricts the correlation filter to parts suitable for improving search range and performance for irregularly-shaped objects. In CSR-DCF, the default positive sample is clean and there is no specific sample purification strategy for dealing with sample contamination. Therefore, this method might result in drift when the target undergoes serious occlusion. Danelljan et al. [30] proposed to use spatially-regularized discriminative correlation filters (SRDCF) to mitigate unwanted boundary effects. Similarly, this model has still not introduced sample reliability analysis, and the default training samples are reliable and uncorrupted. Bibi et al. [31] proposed to jointly lean the filter and target responses for alleviation of fast motion and boundary effects. They first raise doubt about the reliability of the assumption whose sample set obeys single-centered Gaussian Distribution and point out that this assumption is invalid when target moves fast. However, they do not consider contamination of the sample, so there is still drift when the target is seriously occluded. Different from the above methods, this paper’s proposed method considers both sample reliability and boundary effects. In addition, the proposed approach uses a unified framework to solve both of these problems simultaneously.

## 3. Proposed Method

### 3.1. Standard Discriminative Correlation Filter

The standard discriminative correlation filter (DCF) for tracking is trained by using a number of grayscale image patches $x_{1},x_{2}$, …, $x_{t}$ of size M×N and their desired correlation outputs g. New variation of the optimal filter attained from the latest patch is obtained by minimizing the objective function over h:

(1) $$\underset{h_{t}}{\min}\underset{m,n}{\sum }{\left\|P^{m}(w_{s}\cdot x_{t})P^{n}\cdot h_{t}-g\left(m,n\right){\|}_{2}^{2}+\lambda \|h_{t}\right\|}_{2}^{2}.$$

Here, denotes point-wise multiplication. Matrix P is the permutation matrix that cyclically shifts a matrix, and $P^{m}xP^{n}$ means shifting the rows of matrix x by m and shifting the columns of matrix x by n. Variables (m,n)∈{0,…,M−1}×{0,…,N−1} are the shift quantities and λ controls the amount of regularization. The spatially weighted map $w_{s}$ is often chosen to be a cosine window [18,21] or a Gaussian window [32] to alleviate noises in Fourier representation and reduce the influence of background pixels. Using Parseval’s formula, (1) can be transformed into the Fourier domain and the solution to Equation (1) in the Fourier domain is

(2) $$H_{t}=\frac{\bar{G}\cdot F_{t}}{{\bar{F}}_{t}\cdot F_{t}+\lambda },$$

where capital letters denote the discrete Fourier transformations (DFTs) of the corresponding functions and $F_{t}$ represents the DFT of $w_{s}\cdot x_{t}$. The bar represents complex conjugation. Note that (2) only considers one training sample. An optimal filter can be achieved by minimizing the error of all training samples [33]. However, this solution is very time consuming, thus, many trackers [17,18,32] use the following updated strategy to get a robust approximation of the optimal filter:

(3) $$A_{t}=\left(1-\eta \right)A_{t-1}+\eta \bar{G}\cdot F_{t}$$

(4) $$B_{t}=\left(1-\eta \right)B_{t-1}+\eta \left(\bar{F_{t}}\cdot F_{t}+\lambda \right)$$

(5) $$H=A_{t}/B_{t},$$

where η is the learning rate and/denotes element-wise division. $A_{t}=\bar{G}\cdot F_{t}$ and $B_{t}=\bar{F_{t}}\cdot F_{t}+\lambda$.

Given an image patch of size M×N in the next frame, the confidence scores are computed as

(6) $$\hat{y}={ℱ}^{-1}\left\{\bar{H}\cdot F_{t+1}\right\},$$

where ${ℱ}^{-1}$ denotes the inverse DFT operator and $F_{t+1}$ represents the DFT of $w_{s}\cdot x_{t+1}$. The new target location is estimated to be at the maximum confidence score of $\hat{y}$.

### 3.2. Consistently Sampled Correlation Filters

**Figure 1:**
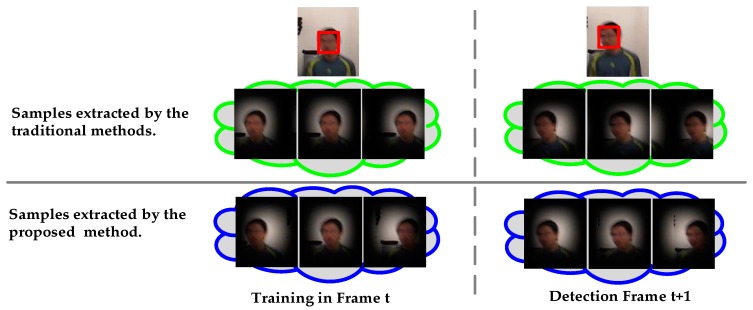
Sampling strategies of traditional correlation filter-based methods and the proposed method. The boy in the figure is blurry due to fast motion. Two input images where the red box area is the target (**Top row**). Weights and image content execute synchronously cyclic shifts (**Middle row**). Weights are independent and do not execute cyclic shifts with image content (**Bottom row**).

Traditional correlation filter-based methods have a common shortcoming, as illustrated by the green cloud in middle row of Figure 1. They all use cyclic shifts of the image patches as samples, which first weight the image patch with a fixed weight map (i.e., hamming window) and then use all cyclic shifts of the image patch as samples. This may result in a set of redundant training samples and inconsistencies between training and detection samples. In addition, these methods cannot handle large search regions because large search regions contain too much background information, resulting in imbalances between foreground information and background information in a sample.

We constructed a consistent sample constraint strategy consisting of spatially-consistent and temporally-consistent samples to alleviate the redundancies in training samples caused by cyclical shifts and to eliminate the inconsistencies between training samples and detection samples. Compared with traditional sampling strategies, our method does not bind the weight and image patches together. Instead, we make every training sample have its own weight map. This strategy alleviates redundancies in training samples which are caused by cyclical shifts and eliminates the inconsistencies between training samples and detection samples.

#### 3.2.1. Spatially-Consistent Sampling in Training Steps

In order to collect a consistently weighted training set, we gave each shift of the training image patch a weighted function rather than just the image patch. Hence, we change the objective function to be:

(7) $$\underset{h_{t}}{\min}\underset{m,n}{\sum }{\left\|\left(w_{s}\cdot \left(P^{m}x_{t}P^{n}\right)\right)\cdot h_{t}-g\left(m,n\right)\right\|}_{2}^{2}+\lambda {\left\|h_{t}\right\|}_{2}^{2}.$$

It is easy to find that putting a weight function on every sample has the same effect as putting the weight function on the filter h in the first term:

(8) $$\underset{h_{t}}{\min}\underset{m,n}{\sum }{\left\|\left(P^{m}x_{t}P^{n}\right)\left(w_{s}\cdot h_{t}\right)-g\left(m,n\right)\right\|}_{2}^{2}+\lambda {\left\|h_{t}\right\|}_{2}^{2}.$$

By applying Parseval’s theorem to (8), the filter h can also be obtained by minimizing the following objective function over $H_{t}$ in the Fourier domain:

(9) $$\underset{H_{t}}{\min}{\left\|X_{t}\cdot \left(\frac{W_{s}}{MN}*H_{t}-G\right)\right\|}_{2}^{2}+\lambda {\left\|H_{t}\right\|}_{2}^{2},$$

where ∗ denotes circular convolution.

To solve this optimization problem, we vectorize all scalar valued functions in Equation (9). After transforming point-wise multiplication and circular convolution into a matrix product, we then get:

(10) $$\underset{H_{t}}{\min}{\left\|D\left(X_{t}\right)\left(C\left(W_{s}\right)H_{t}\right)-G\right\|}_{2}^{2}+\lambda {\left\|H_{t}\right\|}_{2}^{2},$$

where $D\left(X_{t}\right)$ represents the diagonal matrix where the diagonal elements are formed by $X_{t}$. The MN×MN matrix $C\left(W_{s}\right)$ is obtained by concatenating the vectorizations of all possible cyclic shifts of $W_{s}/MN$.

#### 3.2.2. Temporally-Consistent Sampling in the Detection Step

To prevent the collection of corrupted detection samples caused by movement of the target object, we separately put the weight function on every shift sample in the detection step. Thus the confidence scores $\hat{y}$ are obtained by:

(11) $$\hat{y}\left(m,n\right)=\left(w_{s}\cdot \left(P^{m}zP^{n}\right)\right)\cdot h=\left(P^{m}zP^{n}\right)\cdot \left(w_{s}\cdot h\right),$$

where z is the M×N feature map extracted from the next frame for detection. Using the convolution theorem, all MN computations in (11) can be achieved by computing only once in the Fourier domain, then transforming them back into the spatial domain using the inverse Discrete Fourier Transform method. Thus we obtained the responses at all positions by

(12) $$\hat{y}=F^{-1}(D(Z)C(W_{s})\hat{H}),$$

where Z is the vectorization of the DFT of z. The new target location is estimated to be at the maximum confidence score of $\hat{y}$.

### 3.3. Anisotropic Spatially-Regularized Correlation Filters

**Figure 2:**
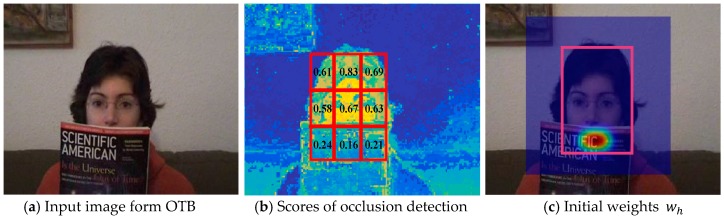
Constructing anisotropic weight constraints based on occlusion detection.

Traditional DCF-based tracking models assume that the target region used for training is not occluded. Under this assumption, the estimated filter’s higher responses correspond to spatial structure features of the target while its lower responses match with the spatial structure of the background region. However, when the target is occluded, the corresponding parts of the estimated filter should not have high responses anymore, but traditional methods did not notice this. In this case, the filter’s discrimination may be reduced and even lead to tracking drift. In order to purify the filter, we proposed to study an anisotropic spatially-regularized correlation filter based on prior information on occlusion detection. Combined with the consistently-sampled correlation filters proposed in Section 3.2, we constructed a unified DCF model:

(13) $$\underset{h_{t}}{\min}\underset{m,n}{\sum }{\left\|\left(w_{s}\cdot \left(P^{m}x_{t}P^{n}\right)\right)\cdot h_{t}-g\left(m,n\right)\right\|}_{2}^{2}+\lambda {\left\|w_{h}\cdot h_{t}\right\|}_{2\;}^{2}$$

where $w_{h}$ is an anisotropic spatially-weighted matrix obtained by occlusion detection. The anisotropy is determined by the location of the occluded region. In [34], Meshgi et al. proposed to detect occlusion through studying foreground probability distributions, where the target is divided into grid cells and the likelihood of occlusion is determined for each cell in a data-driven fashion. Motivated by [34], we adopted a strategy based on grid cells to evaluate occlusion. The difference with [34] is that we used a simpler and faster target–background color statistical distribution [24] to measure occlusion confidence for every cell. Figure 2a briefly illustrates the construction of the $w_{h}$. Figure 2b shows a statistical graph of target scores where the target region is divided into nine equal-sized cells, and cells with a score of less than 0.5 are considered to be occluded.

Specifically, scores are estimated by differences in the statistical distribution of colors between target and background, which indicate the possibility that the current patch belongs to the target. Referring to [24], we employed a color histogram-based Bayes classifier to calculate the object color statistical distribution of every pixel, which is described as:

(14) $$p\left(x\in O|O,S,b_{x}\right)=\{\begin{array}{cc} \frac{H_{O}^{I}\left(b_{x}\right)}{H_{O}^{I}\left(b_{x}\right)+H_{S}^{I}\left(b_{x}\right)} & if\;I\left(x\right)\in I\left(O\cup S\right)\\ 0.5 & otherwise \end{array}$$

where O denotes a rectangular object region and S is the surrounding region of O. $H_{O}^{I}\left(b_{x}\right)$ denotes the *b*-th bin of the non-normalized histogram *H* computed over the region O∈I and x represents pixel position in image I. Additionally, $b_{x}$ denotes the bin assigned to the color components of *I*(*x*). In order to obtain a more reliable target description, we chose to correct the model with the first frame when updating the model:

(15) $$p\left(x\in O|O,S,b_{x}\right)=\lambda_{p}p\left(x\in O|O_{1},S_{1},b_{x}\right)+\left(1-\lambda_{p}\right)p\left(x\in O|O,S,b_{x}\right),$$

Then, the score of every cell is calculated as:

(16) $$Score\left(cell_{i}\right)=\frac{\sum_{x\in cell_{i}}p\left(x\in O|O,S,b_{x}\right)}{\left|cell_{i}\right|},$$

where $\left|cell_{i}\right|$ denotes the sum of all the pixel values in *i*-th cell. Next, we merged all adjacent occlusion cells and chose the biggest connected region as the current occlusion region to construct the $w_{h}$. Figure 2c is the visualization of the spatial distribution of $w_{h}$, where hotter colors correspond to greater weight and the colored area is the search area. The $w_{h}$ obeys two-dimensional Gaussian distribution centered at the center of the occlusion area and its variances are constrained by the length and width of the occlusion area. The computed Gaussian distribution weight map is only applied to the occlusion area in $w_{h}$ and the weights in the remaining area in $w_{h}$ are all set to 0. The Equation (13) can be converted into Equation (17) by the Fourier transform based on Parseval’s theorem. Therefore, the filter $h_{t}$ can also be obtained by minimizing the Equation (17) over $H_{t}$ in the Fourier domain:

(17) $$\underset{H_{t}}{\min}{\left\|X_{t}\cdot \left(\frac{W_{s}}{MN}*H_{t}-G\right)\right\|}_{2}^{2}+\lambda {\left\|\frac{W_{h}}{MN}*H_{t}\right\|}_{2}^{2},$$

Similar to the transformation from Equation (9) to (10), we can change Equation (17) to the following objective function:

(18) $$\underset{H_{t}}{\min}{\left\|D\left(X_{t}\right)\left(C\left(W_{s}\right)H_{t}\right)-G\right\|}_{2}^{2}+\lambda {\left\|C\left(W_{h}\right)H_{t}\right\|}_{2}^{2},$$

### 3.4. Solutions to the Proposed CSSAR Problem

To ensure faster convergence, we reformulated (18) to an equivalent real-valued optimization problem using the method proposed in [30]. We then got:

(19) $$\underset{H_{t}}{\min}{\left\|D_{t}\left(C_{s}{\tilde{H}}_{t}\right)-\tilde{G}\right\|}_{2}^{2}+\lambda {\left\|C_{h}\left({\tilde{H}}_{t}\right)\right\|}_{2}^{2},$$

where $D_{t}=BD\left(X_{t}\right)B^{H}$, $C_{S}=BC\left(W_{s}\right)B^{H}$, $\tilde{G}=BG$, $C_{h}=\frac{1}{MN}BC\left(W_{h}\right)B^{H}$, and ${\tilde{H}}_{t}=BH_{t}$. The unitary matrix B is a transformation matrix, which transforms the complex-valued matrix into a real-valued matrix and preserves the Hermitian symmetry for ensuring faster convergence.

We proposed two solutions to solve the optimization problem with (19). First, since the objective function in (19) is convex, the closed-form solution was achieved by setting the derivative of Equation (19) to be zero:

(20) $$\frac{\partial f({\tilde{H}}_{t})}{\partial {\tilde{H}}_{t}}=-{\left(D_{t}C_{s}\right)}^{T}\tilde{G}+({\left(D_{t}C_{s}\right)}^{T}\left(D_{t}C_{s}\right)+\lambda C_{h}){\tilde{H}}_{t}=0.$$

Let $P_{t}={\left(D_{t}C_{s}\right)}^{T}\left(D_{t}C_{s}\right)+\lambda C_{h}$ and $Q_{t}={\left(D_{t}C_{s}\right)}^{T}\tilde{G}$, (18) can be simplified as:

(21) $$P_{t}{\tilde{H}}_{t}=Q_{t}.$$

Then the filter was obtained by ${\tilde{H}}_{t}=P_{t}^{-1}Q_{t}$.

However, large matrix operations are often time consuming, so we explored the sparsity of $W_{s}$ and $W_{h}$ by setting those elements which are smaller than a threshold to be zero. Then we obtained a sparse matrix, $P_{t}$. Thus, we reduced the computations of large matrix operations in (20) and (21). Note that (21) only considers one training sample. To get an efficient online tracker, we used a strategy similar to Equations (3)–(5) to get a robust approximation of the optimal filter:

(22) $$P_{t}=\left(1-\eta \right)P_{t-1}+\eta \left({\left(D_{t}C_{s}\right)}^{T}\left(D_{t}C_{s}\right)+\lambda C_{h}\right)$$

(23) $$Q_{t}=\left(1-\eta \right)Q_{t-1}+\eta {\left(D_{t}C_{s}\right)}^{T}\tilde{G}$$

(24) $${\tilde{H}}_{t}=P_{t}^{-1}Q_{t}$$

where η is the learning rate.

However, although we explored the sparsity of $W_{s}$ and $W_{h}$, (24) was still computationally very demanding. We only used (24) to initialize the filter for the iteration method described below. To design an efficient method, we solved the normal Equation (21) by the Gauss-Seidel iteration. The Gauss-Seidel method decomposes the matrix $P_{t}$ into a lower triangular component $L_{t}$ and a strictly upper triangular component $U_{t}$ such that $P_{t}=L_{t}+U_{t}$. The filter was iteratively calculated by the equation:

(25) $$L_{t}{\tilde{H}}_{t}^{\left(i\right)}=Q_{t}-U_{t}{\tilde{H}}_{t}^{\left(i-1\right)}$$

where i=1,2,…, and denotes the iterations. Equation (25) can be efficiently solved in MATLAB by the triangular solver for sparse matrices. We found that just a few iterations of (25) were enough for achieving an effective updated filter in our tracker. We used the filter computed in the previous frame as the initial iteration of the next frame. For the first frame, we obtained the initial estimation by solving (24). After we get the estimated ${\tilde{H}}_{t}$, we applied ${\hat{H}}_{t}=B^{H}{\tilde{H}}_{t}$ to obtain the DFT coefficients of the filter. Then we could use (12) to estimate the target position in the next frame.

To integrate multiple features, we simply separately calculated each feature channel’s response map and added them together as the final confidence map. We used a coarse grid strategy to construct $x_{t}$ and z for computational efficiency. To refine the confidence scores in sub-grids, we used the polynomial interpolation method near the estimated target location in the coarse grid map $\hat{y}$. In addition, we built a pyramid around the estimated location for scale estimation similar to [21] after we got the estimated location.

## 4. Experiments

We compared the proposed tracker with the 11 state-of-the-art trackers, including MIL [10], OAB [11], TLD [13], CSK [18], DSST [21], BACF [28], SRDCF [30], Struck [35], CXT [36], VTD [37], and DFT [38]. In experiments, test datum of BACF (Available at: http://www.hamedkiani.com/bacf.html) and SRDCF (Available at: http://www.cvl.isy.liu.se/research/objrec/visualtracking/regvistrack/index.html) came from the author’s home page and all other trackers used the source code provided by the author. The parameter settings from authors were kept for all the test sequences. All testing sequences were from the object tracking benchmark (OTB) [39].

### 4.1. Features and Parameters

Our tracker was initialized as follows: We explored the sparsity of the Gaussian function in its Fourier domain by eliminating all discrete Fourier transform (DFT) coefficients which were smaller than a threshold for computational efficiency. In our tracker, we used the Histogram of Oriented Gradient (HOG) feature, which has 27 channels and is extracted by using a cell size of 4×4 pixels. During the tracking process, all samples are resized to size M×N, where we let M=N and restricted M to ensure a maximum size of 55. The size of the search region was set to be $4^{2}$ times the size of the target area. The iterations of the Gauss-Seidel method were set to be 7 and the learning rate was adopted as η = 0.025. All parameters remained fixed in all experiments. The proposed tracker was implemented on MATLAB 2016a. The experiments were performed on a PC with 8 GB RAM and a Intel i7-4790 CPU (3.6 GHz), and the tracker ran at 3 fps.

### 4.2. Qualitative Evaluation

**Figure 3:**
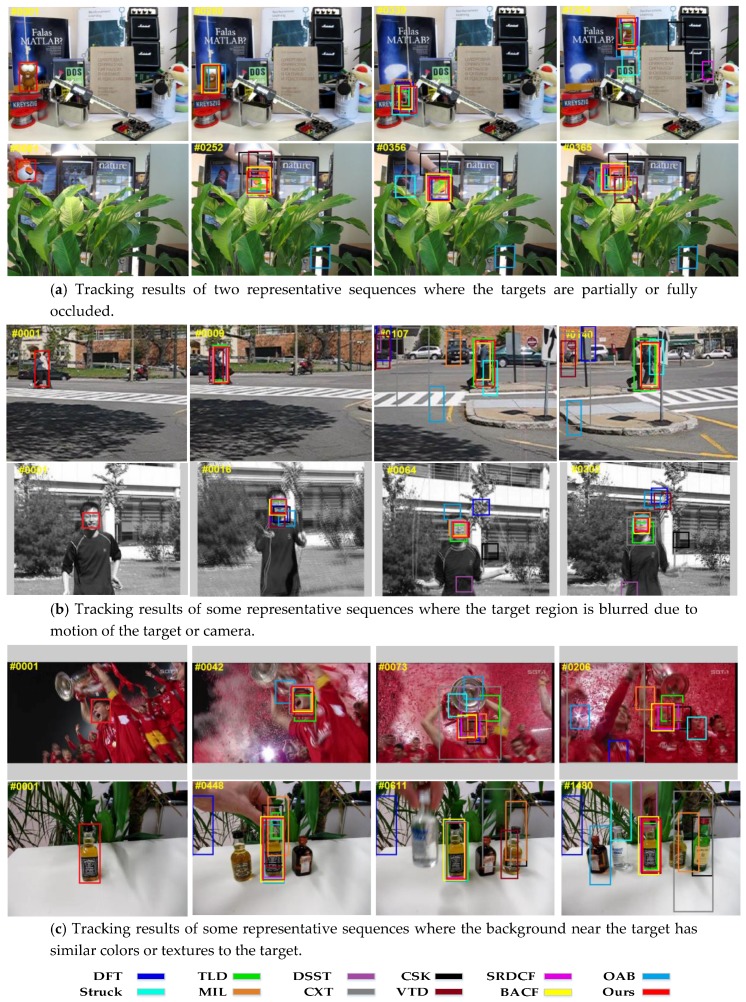
Tracking results of the proposed method and 10 state-of-the-art tracking algorithms for some representative sequences, including occlusion, motion blur, and background clutter.

Figure 3 reports the tracking results of the qualitative evaluation. In the evaluation, we compared the proposed tracker with 11 state-of-the-art trackers for some of the most challenging scenes (i.e., occlusion, motion blur, background clutter) in the OTB.

In Figure 3a, we show two typical scenes where the targets undergo occlusion. The Lemming sequence represents some scenes where the targets undergo full occlusion. In the Tiger2 sequence, the target is occluded partially. Our tracker can effectively track these objects because the uncorrupted parts of the targets and the background context can still form some uncorrupted samples for training and detection. Most of the training and detection samples of the traditional correlation filter based trackers, e.g., DSST and CSK, were corrupted (not consistently sampled). In addition, the sizes of their search areas were limited. Thus the trackers may drift after occlusion or fail to track the targets. Struck and OAB can handle some slight occlusions, but when undergoing severe occlusion they all drift away. Compared with Struck and VTD, SRDCF, which has a larger search area and can deal with more severe occlusions, cannot handle full occlusion (i.e., the Lemming sequence). However, our tracker, which is constrained by the spatial anisotropic regularization term during training, can effectively deal with full and transient occlusion.

In Figure 3b, we selected two representative sequences where the target region is blurred. In the Couple sequence, the target region is blurred because of the motion of camera. In the Jumping sequence, the target region is blurred because of the fast motion of target. The CSK and DSST worked well in the beginning frames of Couple sequence but failed later. Compared with the CSK and DSST, the proposed CSSAR tracker, which allows for a larger search region than CSK and DSST due to its consistent sampling strategy, performed well in these two scene-types. The TLD tracker can redetect the target after tracking failure, but it poorly handles situations when targets undergo both deformation and abrupt motion. Struck is susceptible to abrupt motion because of the sampling strategy. Additionally, BACF demonstrated better results than our CSSAR in the Couple sequence where the target is not only blurred, but the background is also cluttered. BACF exploits all background patches as negative examples for learning a filter, which can improve its ability to distinguish the target from background clutter.

Figure 3c shows that the proposed tracker handles background clutter well. In this figure, we also exhibited two other representative sequences: the Soccer and Liquor sequences. In the Soccer sequence, the background near the target has the similar colors to the target. In the Liquor sequence, the background near the target has similar texture features to the target. The proposed CSSAR tracker gained state-of-the-art results. It uses a consistent sampling strategy where every training sample has its own weight map. The weight map effectively penalizes the background region by assigning it higher weights and increases the discriminative power of the tracker. BACF also gained good results in the two sequences. It exploits real background patches, together with the target patch, to learn the tracked target instead of shifted patches, which can effectively improve the differences between positive and negative samples. CSK and DSST use cyclical shifts to obtain the training samples, which fails to capture the true image content because periodic assumption leads to a limited set of negative training samples. Additionally, this sampling strategy results in imbalances between foreground information and background information when the background features are similar to the target features and close to the target area. Other trackers, i.e., TLD, MIL, and OAB, are susceptible to background clutter because of their random sample strategy. Additionally, SRDCF also demonstrated excellent performance in these two types of scene. It penalizes the correlation filter coefficients corresponding to background area by a spatial regularization component, which significantly mitigates the emphasis on background information in the learned filter and makes the model more discriminative in distinguishing the target from background clutter.

**Figure 4:**
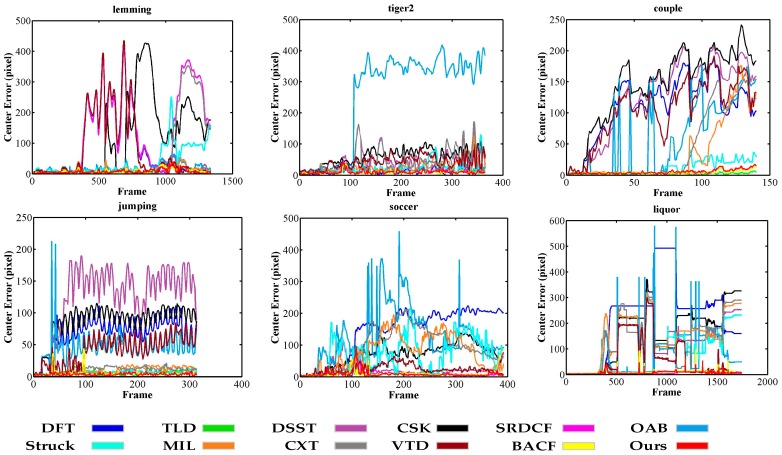
Frame-by-frame comparison of six representative sequences in Figure 3 based on center location errors (in pixels).

In addition, we compared the center location error frame-by-frame for the 6 sequences in Figure 4, which showed that our tracker is able to track targets stably and accurately. In particular, on the Soccer and Liquor sequences, our tracker drifted slightly in the 101–105th and 1034–1040th frames respectively due to severe occlusion or fast in-plane rotation, but it returned to accurate tracking of the targets shortly after. This is because the tracker is constrained by space anisotropic regularization, which saves color information on the target. Table 1 reports average entry location errors for each compared tracker for the 6 sequences. In 3 of the 6 sequences (the Lemming, Jumping, and Soccer sequences), our approach achieved the best results. In two of the remaining three sequences (the Liquor and Tiger2 sequences), our approach obtained the second-best results and were very close to the best ones. Generally, our method performed well against existing trackers.

### 4.3. Quantitative Evaluation

We applied One-Pass Evaluation (OPE), which is a traditional evaluation method used in the OTB. Two criteria, namely the Center Location Error (CLE) and Average Overlap Rate, which is an overlap score from the Pascal VOC overlap ratio (VOR), were employed for quantitative evaluations. The CLE is a widely used measure for evaluating tracking performance. CLE calculates the distance between the center coordinates of the bounding box and the ground-truth. Precision is defined as the percentage of the CLE results below a defined threshold. In practice, the threshold is set to 20 (pixels) to calculate the precision rate of OPE. VOR is defined by $\mathrm{area}\;\left(B_{e}\cap B_{g}\right)/\mathrm{area}\left(B_{e}\cup B_{g}\right)$, where $B_{e}$ is the estimated bounding box and $B_{g}$ is the ground-truth bounding box. In testing we used the area under the curve (AUC) to calculate the success rate of OPE.

**Figure 5:**
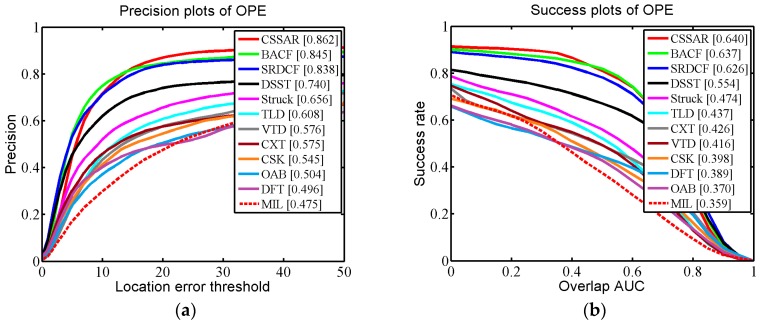
(**a**) reports the average distance precision score at 20 pixels for each method and (**b**) reports the success rate score calculated by the AUC on OTB which consists of 51 videos.

**Figure 6:**
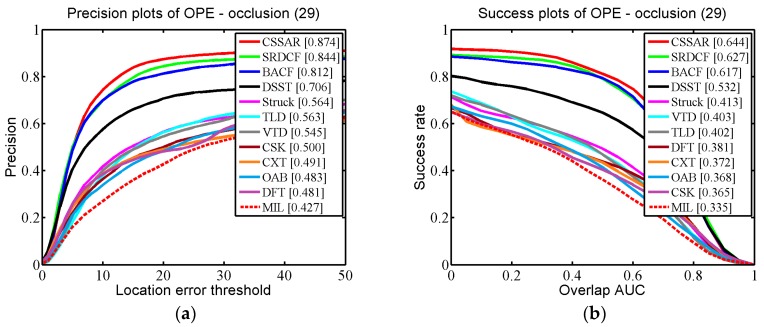
(**a**,**b**) respectively report precision and success plots on some representative sequences where the targets are partially or fully occluded.

**Figure 7:**
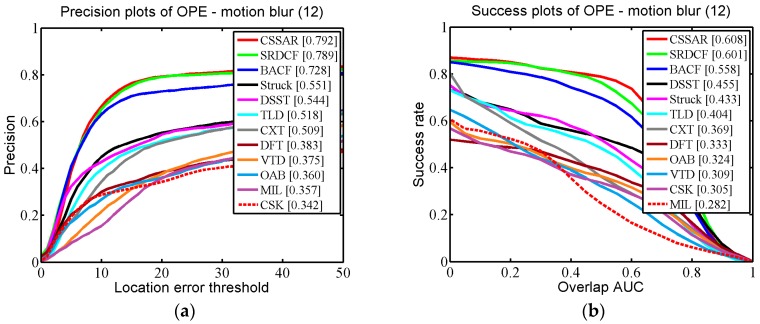
(**a**,**b**) respectively report precision and success plots on some representative sequences where the target region is blurred due to the motion of target or camera.

**Figure 8:**
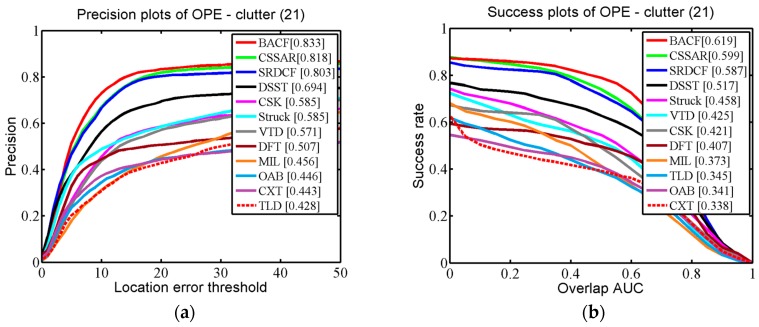
(**a**,**b**) respectively report precision and success plots on some representative sequences where the background near the target has the similar colors or textures to the target.

In Figure 5, the quantitative evaluation results of the proposed tracker and the 11 competing trackers are demonstrated for 51 challenging OTB scenarios. It can be seen that our tracker performed favorably against the 11 state-of-the-art methods (our tracker gains 86.2% in CLE and 64.0% in VOR). Specifically, compared with the second best algorithm, BACF, our tracker was 1.7% (in CLE) and 0.3% (in VOR) higher. The third best tracker, SRDCF, was 2.4% (in CLE) and 1.4% (in VOR) lower than our tracker. Additionally, we specifically describe how our proposed approach outperforms the other trackers in three challenging scenarios (occlusion, background clutter, and motion blur) in detail. In Figure 6, we selected 29 OTB sequences for demonstrating the performance of our algorithm in dealing with the occlusion problem. In these sequences, the targets undergo partial or short-term total occlusion. In this test, the performance of our tracker was higher than the other 11 trackers (our tracker is 87.4% in CLE and 64.4% in VOR), which demonstrates that the space anisotropic regular constraints based on occlusion detection are effective in alleviating occlusion. BACF effectively uses background information to improve discrimination between positive and negative samples, which also can solve slight occlusion. However, when severe occlusion occurs, it may fail to track the target. In Figure 7, there are a total of 12 sequences used for verifying the performance of our tracker in motion blur scenes. Figure 7 shows that our algorithm achieved almost the same excellent performance as SRDCF (our tracker gets CLE scores of 79.2% and VOR scores of 60.8%). Figure 8 reports the results of the 12 trackers in background clutter scenes. There are total 21 sequences in which the targets suffer background clutter. As shown in Figure 8, BACF gained the best results (83.3% in CLE and 61.9% in VOR), benefitting from reliable negative samples. These negative samples are collected from real background patches, which can effectively improve the tracker’s robustness when background clutter occurs. Our algorithm achieved the second best performance (81.8% in CLE and 59.9% in VOR) and is better than SRDCF (our tracker was 1.5% higher than SRDCF in CLE and 1.2% better than SRDCF in VOR).

## 5. Conclusions

This paper presented a novel correlation filter-based online tracking algorithm. On one hand, a spatially- and temporally-consistent sampling strategy was constructed to alleviate redundancies in training samples caused by cyclical shifts and eliminate the inconsistencies between training samples and detection samples. On the other hand, an anisotropic regular constraint was introduced to account for occlusion. Based on these two strategies, our method can simultaneously and effectively deal with large search regions and severe occlusion. Qualitative and quantitative experimental results show that our approach outperforms many state-of-the-art methods.

## Figures and Tables

**Table 1:** Comparison of results in terms of average entry location errors (in pixels). In descending order, the best three estimates are marked in red, blue, and green fonts.

Sequences	DFT	TLD	DSST	CSK	SRDCF	OAB	Struck	MIL	CXT	VTD	BACF	Ours
*Lemming*	77.75	15.74	81.89	114.2	134.5	18.05	37.75	12.06	61.39	79.22	9.170	8.130
*Tiger2*	12.22	73.16	41.45	59.56	11.62	252.7	21.64	27.17	41.44	40.88	8.660	9.220
*Couple*	108.6	2.540	125.2	144.6	3.970	57.62	11.33	34.53	41.76	104.3	4.110	5.140
*Jumping*	67.08	5.940	125.5	85.97	4.470	46.35	6.550	9.990	9.990	41.39	4.830	3.320
*Soccer*	139.5	136.2	20.25	70.51	10.83	127.5	71.36	77.85	89.22	23.56	10.28	7.910
*Liquor*	221.1	55.95	98.53	160.6	4.730	71.07	90.99	141.9	131.8	60.17	9.010	7.210
**Average**	104.4	48.26	82.12	105.9	28.35	95.55	39.94	50.58	62.60	58.25	7.677	6.822
